# Measurement of Retinal Microvascular Blood Velocity Using Erythrocyte Mediated Velocimetry

**DOI:** 10.1038/s41598-019-56239-8

**Published:** 2019-12-27

**Authors:** Breanna M. Tracey, Lakyn N. Mayo, Christopher T. Le, Victoria Y. Chen, Julian Weichsel, Corinne Y. Renner, Jiaqi Li, Joby J. Tsai, Sachin P. Kalarn, Michael T. Ou, Luke Y. Chang, Taariq K. Mohammed, Isa S. K. Mohammed, Khelly A. Shah, Ginger M. Thompson, Anja M. K. Jones, Lily T. Im, Mona A. Kaleem, Osamah J. Saeedi

**Affiliations:** 1 University of Maryland School of Medicine, Baltimore, Maryland United States; 2 UCSF School of Medicine, San Francisco, California United States; 3 Heidelberg Engineering GmbH, Heidelberg, Germany; 4 Donald and Barbara Zucker School of Medicine at Hofstra/Northwell, Hempstead, New York United States; 5 The Johns Hopkins University School of Medicine, Baltimore, Maryland United States; 6 Medical College of Georgia, Augusta, Georgia United States; 7 Rutgers New Jersey Medical School, Newark, New Jersey United States

**Keywords:** Optic nerve diseases, Translational research, Imaging and sensing

## Abstract

Changes in retinal blood flow may be involved in the pathogenesis of glaucoma and other ocular diseases. Erythrocyte mediated velocimetry (EMV) is a novel technique where indocyanine green (ICG) dye is sequestered in erythrocyte ghosts and autologously re-injected to allow direct visualization of erythrocytes for *in vivo* measurement of speed. The purpose of this study is to determine the mean erythrocyte speed in the retinal microvasculature, as well as the intravisit and intervisit variability of EMV. Data from 23 EMV sessions from control, glaucoma suspect, and glaucoma patients were included in this study. In arteries with an average diameter of 43.11 µm ± 6.62 µm, the mean speed was 7.17 mm/s ± 2.35 mm/s. In veins with an average diameter of 45.87 µm ± 12.04 µm, the mean speed was 6.05 mm/s ± 1.96 mm/s. Intravisit variability, as measured by the mean coefficient of variation, was 3.57% (range 0.44–9.68%). Intervisit variability was 4.85% (range 0.15–8.43%). EMV may represent reliable method for determination of retinal blood speed, potentially allowing insights into the effects of pharmacologic agents or pathogenesis of ocular diseases.

## Introduction

Numerous systemic and ocular diseases have been associated with alterations in retinal blood flow (RBF)^1,2^. Local changes in RBF are implicated in the pathogenesis of major causes of blindness such as diabetic retinopathy and glaucoma^3,4^. Furthermore, retinal vasculature is readily accessible for imaging, allowing a window into alterations of blood flow associated with systemic and neurodegenerative diseases such as Alzheimer’s dementia^5^. While structural changes in retinal vasculature, such as change in vessel caliber or increased tortuosity, are established markers of hypertension^6^, diabetes^7^, vasculitis^8^, and neurodegenerative diseases^9^, changes in ocular blood flow have also been linked to these diseases^10^. Dynamic changes in RBF in ocular and systemic disease^11^ may precede these known structural changes, making RBF an important physical biomarker.

Numerous methods to measure RBF have been developed, but there is no established gold standard^12^ for the measurement of RBF in individual vessels *in vivo* that is accurate, precise, and capable of determining flow of multiple vessels over a wide field of view. Color doppler imaging, laser speckle imaging^13^, and more recently OCT angiography^14^ allow for the measurement of relative blood velocity and flow, but not absolute flow. Non-invasive techniques that record RBF in absolute terms include laser doppler imaging and the retinal function imager (RFI), whose coefficients of variation range from 10–11% for the RFI^15,16^ to 20% for laser doppler imaging using the Canon Laser Blood Flow Meter^17^. Adaptive Optics - Scanning Laser Ophthalmoscopy (AO-SLO) also allows for quantification of flow in the microvasculature, but in a relatively small field of view^18^. Furthermore, there are few comparison studies of different devices that measure RBF. The current gold standard for RBF measurement uses fluorescent and radioactive microspheres in animal models^19^. However, this method cannot be easily transferred to humans *in vivo*, and radioactive microspheres similarly have a coefficient of variation of 15–50%^20^. There is a critical need for an accurate and highly reproducible gold standard for human RBF quantification to validate devices and to establish the use of ocular microvascular blood flow as a robust biomarker for ocular and systemic disease.

Erythrocyte mediated angiography (EMA) is a technique in which indocyanine green (ICG) dye is sequestered in the erythrocytes to allow direct visualization of erythrocytes *in vivo*^21^. Prior work has emphasized its role in characterizing vasomotion in the eye^22^. Here, we describe methods used to quantify microvascular erythrocyte speed *in vivo* using a novel variant: Erythrocyte Mediated Velocimetry (EMV). We assess the precision of erythrocyte speed through intravisit and intervisit variability of EMV speed measurements in small arterioles and venules using a commercially available scanning laser ophthalmoscope, which was specifically modified for this purpose.

## Methods

### Participants

**Figure 1:**
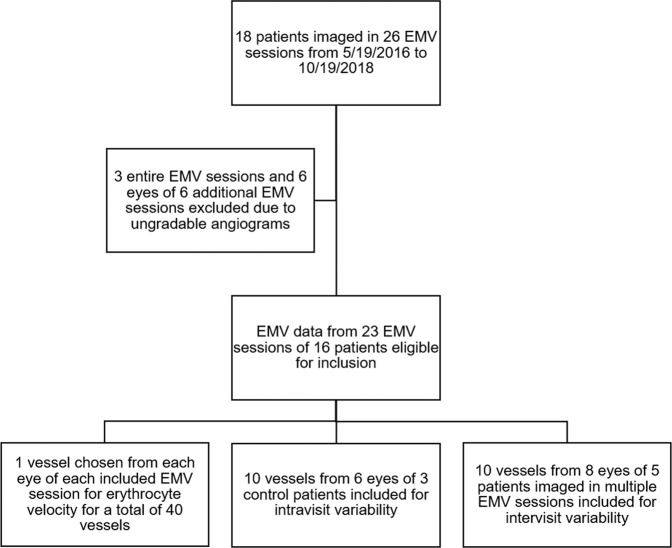
Flowchart showing inclusion and exclusion of patients.

We enrolled glaucoma, glaucoma suspect, and control patients at least 40 years of age. The diagnosis of each patient was determined by a fellowship-trained glaucoma specialist. The diagnosis for glaucoma was made using the preferred practice patterns of the American Academy of Ophthalmology, specifically evidence of characteristic optic nerve damage characterized by retinal nerve fiber layer structural abnormalities or reliable and reproducible visual field abnormalities^23^. Glaucoma suspect was assigned to patients with normal visual field testing and either consistently elevated intraocular pressure or a suspicious-appearing optic disc^24^. Twenty-six EMV sessions were conducted on 35 eyes of 18 individual patients from May 19^th^, 2016 to October 19^th^, 2018. As shown in Fig. 1, we included data from 23 EMV sessions of 29 eyes of 16 individual patients. EMV sessions were considered ungradable if erythrocytes could not be resolved in the vessels for tracking. This was due to media opacity such as dry eye or cataract, or excessive eye movement which precluded image registration. Intravisit variability included only data from control patients. Intervisit variability included data from the 5 subjects who underwent multiple EMV sessions. Blood pressure (BP), pulse (HR), pulse oximetry, intraocular pressure (IOP), keratometry, ocular exam, ocular medications, and co-morbid disease were recorded. Patient demographics are shown in Table 1. Informed consent was obtained from all patients after discussion of the risks and benefits of the study. The study was conducted in accordance with the Declaration of Helsinki, and the study protocol was approved by the institutional review board of the University of Maryland Baltimore.

**Table 1:** Patient Demographics.

Patient	Session (if multiple)	Age	Sex	Race	Diagnosis	Eyes	HR	BP	IOP	
									OD	OS
**1**	a	46	M	Black	Control	OD,OS	64	115/73	14	12
	b	47	M	Black	Control	OD,OS	61	120/69	15	15
**2**	a	55	M	Black	Control	OD,OS	79	143/78	19	20
	b	55	M	Black	Control	OD,OS	80	134/91	21	21
**3**	a	61	M	Black	Glaucoma	OD	66	130/84	15	
	b	62	M	Black	Glaucoma	OD	61	131/84	12	
**4**		65	F	White	Control	OD, OS	82	144/93	17	15
**5**		56	F	White	Glaucoma	OD,OS	62	122/68	14	17
**6**		69	F	Black	Glaucoma Suspect	OD,OS	60	160/86	17	19
**7**		57	F	Black	Glaucoma	OD,OS	64	126/70	21	12
**8**	a	53	M	Black	Glaucoma	OD	75	109/75	11	
	b	54	M	Black	Glaucoma	OD	69	116/69	11	
	c	54	M	Black	Glaucoma	OD	71	117/73	14	
**9**	a	55	F	White	Glaucoma Suspect	OD,OS	50	111/73	16	13
	b	55	F	White	Glaucoma Suspect	OD,OS	54	126/73	15	16
	c	56	F	White	Glaucoma Suspect	OD,OS	62	112/76	11	13
**10**		56	F	Black	Glaucoma Suspect (OD), Glaucoma (OS)	OD,OS	50	103/60	15	38
**11**		60	F	White	Glaucoma Suspect	OD	54	97/50	11	
**12**		57	F	White	Glaucoma	OD,OS	93	137/86	13	14
**13**		66	F	White	Glaucoma Suspect	OD,OS	60	139/82	30	17
**14**		64	F	Black	Glaucoma	OD,OS	71	138/89	20	18
**15**		61	F	Black	Glaucoma Suspect	OD,OS	63	118/56	15	15
**16**		64	F	White	Glaucoma Suspect	OD,OS	68	128/76	18	20

### Erythrocyte preparation

Cell preparation was conducted as described by Flower *et al*.^21,22^. Up to 34 mL of blood was drawn from each subject. Using sterile procedures, erythrocyte ghosts produced from the blood were loaded with ICG dye. Following pupillary dilation with Tropicamide 0.5%, up to 1 mL of autologous ICG-loaded erythrocytes were injected intravenously. These cells persisted and were used to measure erythrocyte speed for up to three hours for one session. As previously described, ICG-loaded erythrocytes have comparable physical properties to native erythrocytes^22^.

### Image acquisition

A Heidelberg Retinal Angiograph (HRA) 2 (Heidelberg Engineering GmbH, Germany) was used to acquire 10–20 second angiograms of subjects undergoing EMV. Angiograms of the disc, macula, and peripapillary retina were obtained in both eyes of all subjects except for one monocular patient. Angiograms were acquired with a custom scan pattern at 24.6 frames per second with a 15-degree horizontal and 7.5-degree vertical field of view. Conventional ICG or fluorescein angiography images were obtained concurrently or at the conclusion of each EMV session.

### Image registration

To account for eye motion, images were registered by aligning the entire angiogram sequence to a reference image. We used a custom MATLAB (MathWorks, version 2018a) script that performed spatial domain registration with algorithms utilizing the overall similarities of the images. The registration script allows for rotation and translation to align the image with a user-selected reference image. No scale change was permitted to preserve the calculated scale. Both the mean squares and Mattes mutual information metrics were used independently to register the images, with the final compiled registered sequence consisting of the optimally registered frames based on either of the two metrics, as selected by the user. This effectively eliminated eye motion and generated a consistent set of coordinates to allow for accurate speed measurement.

### Image registration validation

In addition to visual inspection of registered images, registration was quantitatively validated by comparing the stability between frames in unregistered and registered EMV angiograms. The stationary RBC closest to the optic disc and clearly visualized in all angiogram images was used as a reference point. Motion between frames was quantified by computing the straight-line distance between the stationary RBC on adjacent frames. A straight-line distance of more than three pixels between the stationary RBC coordinates on two adjacent frames was classified as poor stability. The number of frames with poor stability was compared between corresponding unregistered and registered angiogram sequences. To validate image registration, we chose five representative sequences chosen for their varying degrees of eye motion, one with minimal eye motion, one with moderate eye motion, and three with substantial eye motion, as judged by visual inspection. We compared the percentage of frames with poor stability before and after registration.

### Erythrocyte speed measurement

**Figure 2:**
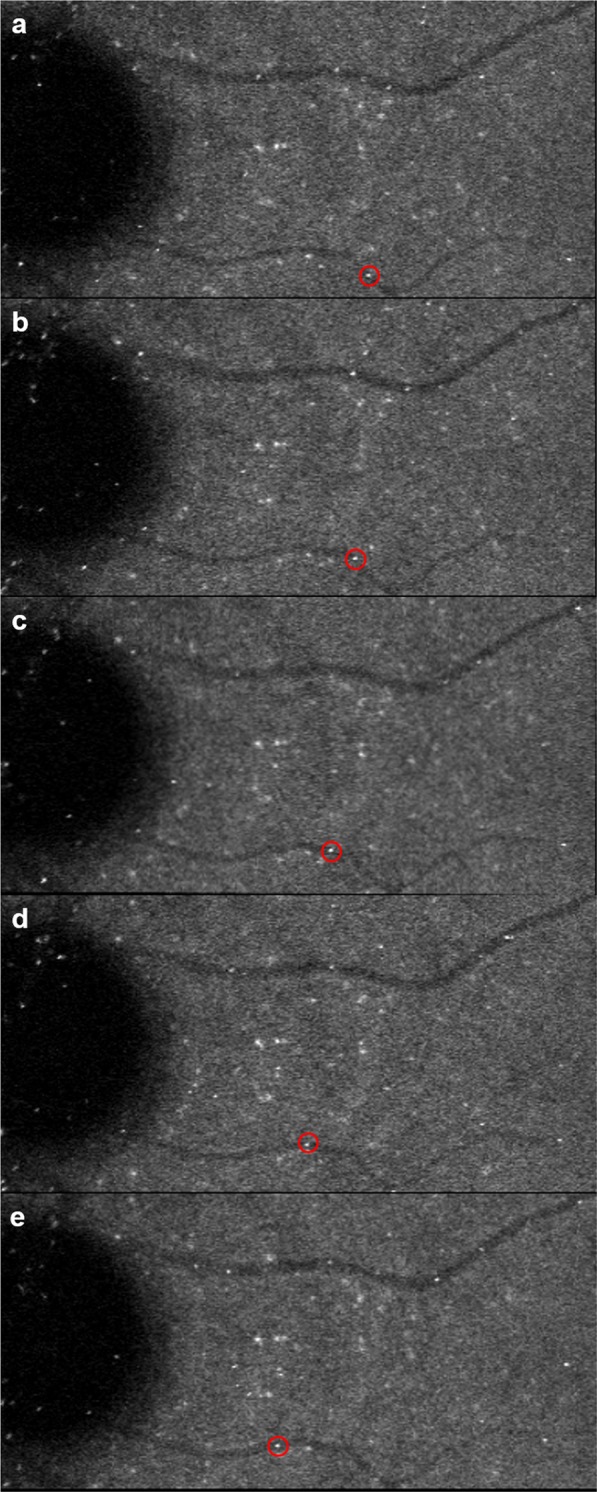
Example of erythrocyte tracking from erythrocyte mediated velocimetry images. Red circles show an individual erythrocyte flowing in a small vein along the retina (**a**–**e**).

**Figure 3:**
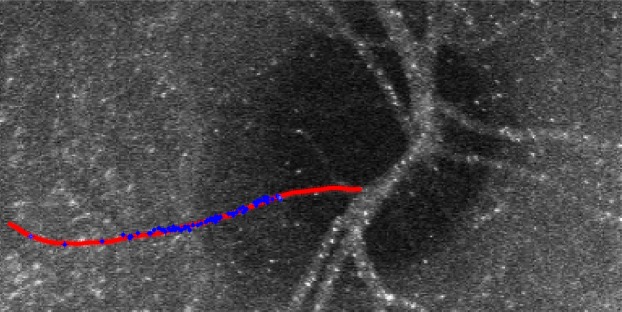
EMV image showing coordinates of many individual erythrocytes from an angiogram sequence (blue) overlaid on user-drawn vessel path (red).

A protocol for speed measurement was developed that consists of manual erythrocyte tracking followed by erythrocyte speed measurement using a custom MATLAB script. Individual erythrocytes were tracked as they flowed in a vessel over multiple EMV image frames (Fig. 2). Tracking was only done when there was clearly one ICG labelled erythrocyte flowing in a vessel segment in a given time without any other ICG labelled erythrocytes that would create uncertainty. To further standardize erythrocyte tracking, cells were eligible for tracking only if the same cell was visible on at least three consecutive frames within the vessel of interest. Only erythrocytes visible on the retina were tracked, and those in the optic disc were excluded. After erythrocyte tracking, speed was determined in MATLAB using a semi-automated custom script. For standardization, the center of the brightest pixel was recorded as the cell location. After recording all cell locations, the user defined the vessel path (Fig. 3). The distance along the vessel course in pixels was converted to microns using the conversion scale provided by the Heidelberg HRA2 software, accounting for keratometry^25^. Speed was calculated using the vessel distance traveled by an erythrocyte over two adjacent frames and dividing by the time between frames. Mean speed was obtained by averaging the individual erythrocyte speed measurements. At least 30 individual measurements were required. Angiograms were only eligible for inclusion if they included at least 85 frames.

For determination of erythrocyte speed, one vessel chosen at random was analyzed per eye for each included EMV session. Due to concerns about the temporal resolution of imaging at 24.6 frames per second, we conservatively chose to exclude arteries over 60 µm and veins over 80 µm. While our imaging technique allows for measurement of erythrocyte speeds up to 25 mm/s, we conservatively chose this criteria for vessel caliber to place our mean speeds less than 15 mm/s^26^. For calculations of intravisit and intervisit variability, to account for variations in the field of view in each angiogram, we ensured the same region of the vessel was analyzed. This vessel region was chosen adjacent to the optic disc and limited to no more than 100 pixels in the horizontal direction, approximately 1 mm. In the case of one macular vessel, the region was chosen to include the terminal end of the vessel, which was best visualized on the angiogram.

### Vessel diameter measurement

The diameter of each vessel was determined from registered sequences of conventional ICG or fluorescein angiograms obtained concurrently or immediately after the EMV imaging session. Conventional ICG images were obtained at 24.6 frames per second with a 15° horizontal and 7.5° vertical field of view. Fluorescein angiography images were obtained at 4.7 frames per second with a 30° horizontal and 30° vertical field of view. Diameter measurements were obtained using a custom MATLAB script to trace vessel boundaries and determine the average diameter over the course of the vessel segment. To account for possible variation in vessel diameter with the cardiac cycle^27^, diameter measurements were obtained on five separate images, each approximately 200 msec apart, and averaged.

### Statistical analysis

Interrater reliability was evaluated with the intraclass correlation coefficient (ICC), two-way mixed, absolute agreement^28^, using 380 speed measurements of two independent graders on a standard set of angiograms. Intravisit variability, or the variability of erythrocyte speed within a single EMV session, was evaluated with 10 vessels (3 arteries and 7 veins) in six eyes of three control patients imaged twice within the same EMV session. We also evaluated intravisit variability in 10 vessels (3 arteries and 7 veins) of five eyes of four glaucoma patients. Intervisit variability, or the variability of erythrocyte speed across time, was evaluated with 10 vessels (3 arteries and 7 veins) of eight eyes of five patients who underwent two EMV sessions separated by at least 60 days. For calculation of intervisit variability, if two angiograms of a given vessel from the same visit were available, the measurements from both angiograms were combined into a single mean speed. If patients had angiograms from three EMV sessions, the two visit days with the highest quality angiograms were used for comparison. The coefficient of variation (CV) was used for determination of intravisit and intervisit variability. The CV is defined as the ratio of the standard deviation (SD) to the mean, as shown in Eq. (1).

1 $$CV=\frac{SD}{mean}$$

The CV was calculated for each vessel individually, using mean speeds obtained from the two angiograms. Mean ocular perfusion pressure (MOPP) was computed for each EMV session using the patient’s systolic blood pressure (SBP), diastolic blood pressure (DBP) and IOP, as shown in Eq. (2)^29^.

2 $$MOPP=\frac{2}{3}(\frac{1}{3}\,\ast \,SBP+\frac{2}{3}\,\ast \,DBP)-IOP$$

Overall mean speed in retinal arteries and veins was determined by averaging mean speeds obtained from individual angiograms from all included EMV sessions. Data is presented as mean ± SD. All statistical analysis was performed in SPSS Statistics (IBM Corporation, version 24.0).

## Results

### Image registration

The five sequences used for quantitative image registration validation exhibited minimal, average, and high eye motion, judged by visual inspection. In the unregistered images, the sequences had a mean of 12.5% frames with poor stability. In the corresponding registered image sequences, the angiograms had a mean of 0.9% frames with poor stability (p < 0.01), effectively validating the MATLAB image registration script.

### Variability of speed

**Figure 4:**
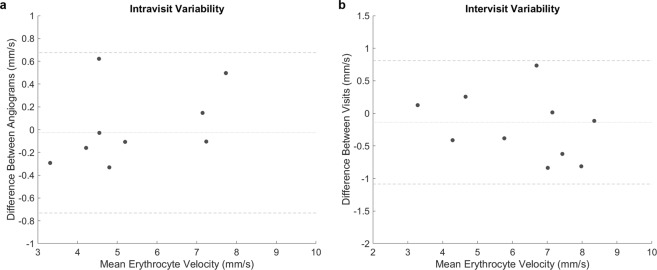
Difference in velocity as a function of the mean velocity between angiograms acquired in the same session (**a**) or separate sessions. (**b**) The center line represents the mean difference in velocity between sessions. The outer lines represent the mean difference ±1.96(SD).

The interrater variability, defined as the ICC for two independent graders calculating erythrocyte speed, is 0.983. The intravisit variability, defined as the average CV between two angiograms obtained in the same EMV session, is 3.57% (range 0.44–9.68%) in control subjects (Tables 2) and 6.66% in glaucoma patients (P > 0.10). The intervisit variability, defined as the average CV between two EMV sessions, is 4.85% (range 0.15–8.43%), as shown in Table 3. The mean time between visits was 220 days. Graphical representation of the intravisit and intervisit variability is shown in Fig. 4.

**Table 2:** Intravisit Speed – Comparison of speed from angiograms obtained within the same EMV session.

Patient	Eye	Vessel	Diameter, µm (SD)	Angiogram #1	Angiogram #2	CV
				Velocity, mm/s (SD)	Velocity, mm/s (SD)	
1^a^	OD	Vein	39.74 (7.27)	4.14 (1.54)	4.30 (1.78)	2.69%
1^a^	OS	Vein	35.79 (5.33)	3.17 (0.76)	3.46 (0.73)	6.24%
1^b^	OD	Vein	34.69 (5.74)	4.54 (1.98)	4.56 (1.37)	0.44%
1^b^	OD	Vein	40.82 (6.57)	4.85 (1.24)	4.23 (1.18)	9.68%
2^a^	OD	Artery	34.34 (6.90)	7.22 (2.19)	7.07 (2.47)	1.45%
2^a^	OD	Vein	36.35 (6.23)	4.63 (1.36)	4.97 (1.82)	4.87%
2^a^	OS	Vein	53.00 (9.77)	5.14 (1.02)	5.25 (1.18)	1.46%
2^b^	OD	Artery	35.05 (6.02)	7.98 (2.83)	7.48 (1.89)	4.54%
4	OD	Vein	29.88 (5.13)	7.19 (1.87)	7.29 (1.61)	1.02%
4	OS	Artery	44.30 (4.51)	10.87 (3.80)	11.39 (5.52)	3.30%
					**Mean CV**	**3.57%**

Superscripts denote patient imaging session (if multiple). CV is defined as the ratio of the standard deviation (SD) to the mean.

**Table 3:** Intervisit Speed – Comparison of speed from angiograms obtained from two separate EMV sessions.

Patient	Eye	Vessel	Diameter, µm (SD)	Visit #1	Visit #2	CV
				Velocity, mm/s (SD)	Velocity, mm/s (SD)	
1^a,b^	OD	Vein	39.74 (7.27)	4.08 (1.63)	4.49 (1.23)	6.80%
1^a,b^	OS	Vein	35.79 (5.33)	3.35 (0.76)	3.22 (1.46)	2.72%
2^a,b^	OD	Artery	34.34 (6.90)	7.12 (2.38)	7.74 (2.43)	5.92%
2^a,b^	OD	Vein	36.35 (6.23)	4.79 (1.60)	4.53 (1.72)	3.88%
2^a,b^	OS	Vein	53.00 (9.77)	5.58 (1.05)	5.96 (1.43)	4.70%
3^a,b^	OD	Vein	38.59 (6.27)	6.60 (1.46)	7.43 (1.70)	8.43%
8^a,b^	OD	Vein	42.92 (7.93)	7.15 (2.42)	7.14 (1.69)	0.15%
8^b,c^	OD	Artery	39.00 (7.80)	7.57 (3.01)	8.38 (2.76)	7.21%
9^a,b^	OS	Vein	67.75 (13.75)	8.29 (3.06)	8.40 (3.75)	0.98%
9^a,c^	OD	Artery	49.62 (9.48)	7.06 (3.86)	6.33 (2.92)	7.76%
					**Mean CV**	**4.85%**

Superscripts denote which patient imaging sessions were compared. CV is defined as the ratio of the standard deviation (SD) to the mean.

### Erythrocyte speed

Of 16 patients, 3 (18.8%) patients had diabetes, 9 (56.3%) had hypertension, 8 (50%) had dyslipidemia, and 6 (37.5%) of patients were on topical glaucoma medications. Using one vessel from each eye, mean erythrocyte speed was calculated in small retinal arteries and veins, as shown in Table 4. MOPP was also calculated for each eye. Eighteen arteries less than 60 µm in diameter and 22 veins less than 80 µm were analyzed. Mean arterial erythrocyte speed was 7.17 mm/s ± 2.35 mm/s in an artery with an average diameter of 43.11 µm ± 6.62 µm. Mean venous erythrocyte speed was 6.05 mm/s ± 1.96 mm/s in a vein with an average diameter of 45.87 µm ± 12.04 µm.

**Table 4:** Erythrocyte speed in small retinal arteries and veins.

Patient	Eye	MOPP	Vessel	Diameter, µm (SD)	Velocity, mm/s (SD)
**Arteries**					
2^a^	OD	47.44	Artery	34.34 (6.90)	7.10 (2.36)
4	OS	58.33	Artery	44.30 (4.51)	10.96 (4.68)
5	OD	43.33	Artery	42.73 (9.27)	7.19 (2.92)
5	OS	40.33	Artery	43.17 (5.50)	6.70 (2.77)
6	OD	54.78	Artery	41.53 (10.36)	7.58 (2.40)
7	OD	38.11	Artery	40.58 (6.62)	7.14 (2.25)
8^b^	OD	45.44	Artery	39.00 (7.80)	7.82 (3.24)
8^c^	OD	44.44	Artery	39.99 (4.27)	8.30 (2.76)
9^a^	OD	41.11	Artery	46.97 (7.53)	5.18 (2.39)
9^b^	OD	45.44	Artery	52.72 (10.96)	6.47 (2.66)
9^c^	OD	47.67	Artery	45.73 ((6.11)	6.21 (2.87)
10	OS	11.56	Artery	49.33 (7.72)	3.77 ((3.81)
11	OD	36.78	Artery	34.81 (5.94)	4.57 (2.75)
12	OD	55.67	Artery	38.50 (6.05)	7.76 (2.95)
12	OS	54.67	Artery	42.78 (7.46)	11.02 (4.47)
13	OD	37.33	Artery	31.18 (1.63)	2.19 (0.89)
14	OD	50.44	Artery	54.72 (12.05)	10.72 ((7.41)
16	OD	44.22	Artery	53.62 (4.65)	8.32 (3.59)
		**Average Arterial Velocity, mm/s (SD)**			**7.17** (**2.35)**
		**Average Arterial Diameter, µm (SD)**			**43.11** (**6.62)**
**Veins**					
1^a^	OD	44	Vein	39.74 (7.27)	4.14 (1.62)
1^a^	OS	46	Vein	35.79 (5.33)	3.32 (0.78)
1^b^	OD	42.33	Vein	40.82 (6.57)	4.82 (1.47)
1^b^	OS	42.33	Vein	38.12 (5.73)	3.21 (1.45)
2^a^	OS	46.44	Vein	53.00 (9.77)	4.42 (1.35)
2^b^	OD	49.22	Vein	34.69 (5.74)	4.53 (1.72)
2^b^	OS	49.22	Vein	49.06 (10.88)	4.49 (1.71)
3^a^	OD	51.22	Vein	38.59 (6.27)	7.51 (1.74)
3^b^	OD	54.44	Vein	38.82 (7.60)	6.61 (1.41)
4	OD	56.33	Vein	29.88 (5.13)	7.15 (1.71)
6	OD	56.78	Vein	43.19 (8.66)	4.79 (1.47)
7	OS	47.11	Vein	42.13 (8.02)	8.49 (3.94)
8^a^	OD	46.56	Vein	42.92 (7.93)	7.20 (2.38)
9^a^	OS	44.11	Vein	67.75 (13.75)	8.01 (3.14)
9^b^	OS	44.44	Vein	71.44 (7.68)	8.42 (3.50)
9^c^	OS	45.67	Vein	68.10 (8.52)	9.00 (3.74)
10	OD	34.56	Vein	44.49 (8.18)	4.50 (2.08)
13	OS	50.33	Vein	40.02 (4.00)	4.17 (1.35)
14	OS	52.44	Vein	68.30 (6.29)	8.57 (4.13)
15	OD	36.11	Vein	40.63 (2.85)	9.01 (4.53)
15	OS	36.11	Vein	40.85 (3.13)	5.61 (1.64)
16	OS	42.22	Vein	40.85 (2.05)	5.18 (2.34)
		**Average Venous Velocity, mm/s (SD)**			**6.05** (**1.96)**
		**Average Venous Diameter, µm (SD)**			**45.87** (**12.04)**

Superscripts denote patient imaging session (if multiple).

## Discussion

We present the results of the first study utilizing EMV, a novel technique, to determine microvascular retinal blood speeds. EMV is a promising and highly precise method for determination of retinal microvascular blood velocity. Variability of quantitative absolute retinal blood flow and blood velocity measurements using other imaging techniques have ranged from 10–11% (RFI)^15,16^ to a mean of 20% (Canon Laser Blood Flowmeter)^17^, and our values were comparable or better.

Retinal velocity measurements obtained using EMV are comparable to previously published velocities using laser doppler^26^ in vessels of similar caliber. For a 42 µm arteriole, Riva, *et al*.^26^ found a mean velocity of approximately 7.5 mm/s, which is similar to 7.17 mm/s found in our study. For a 45 µm venule, mean velocity was approximately 6 mm/s, which is similar to our 6.05 mm/s. A relative advantage of EMV is that while Riva, *et al*. measured velocities in vessels larger than 40 µm in diameter, EMV allows for velocity determination in smaller vessels. Furthermore, by directly measuring the speed of individual erythrocyte ghosts, EMV offers the advantage of quantifying absolute speed. AO-SLO offers a similar ability to quantify flow in the retinal microvasculature, but it does so over a small field a view using equipment that is not commercially available^30^. EMV in comparison is more invasive, but it allows for visualization over a larger field of view with a modified commercially available device.

Despite the more invasive nature of this procedure, we have now imaged 26 patients and shown that it is relatively safe. It may, in fact, be safer than traditional angiography as the aggregate concentration of sequestered ICG dye using EMV is approximately 1/700^th^ that of conventional ICG. Given that the cells are autologously reinjected there is a concern for risk of infection. Sterile technique is strictly adhered to minimize this potential risk. To date, only one patient had an adverse event when they experienced a vasovagal episode after injection of the conventional ICG dye.

This study had broad inclusion criteria, and hence we imaged some patients who did not have gradable angiograms due to media opacity such as cataract or dry eye or due to the learning curve of imaging patients with this new technology. Our broad inclusion criteria resulted in a cohort of patients demographically similar to those at risk for ocular disease with an average age of 57.8 years, and 25% with diagnoses of dry eye or ocular surface disease.

While we chose to assess mean retinal erythrocyte speeds, we note that other metrics such as median, minimum, maximum, range, and distribution may be as important if not more important given the natural physiologic variability of blood flow. Physiologic variability of erythrocyte speed and flow comes from the pulsatile nature of flow, higher in systole and lower in diastole, as well as the distribution of speeds of individual erythrocytes flowing through the vasculature, where cells travelling in the vessel periphery are slower than the cells traveling in the center of the lumen. The potential difference in speed with time and luminal position is why we chose to report mean speeds. The mean speed for each vessel was determined by averaging many individual erythrocyte speeds (mean of 70 individual speed measurements per angiogram) to account for these differences. Given the time-consuming nature of manual speed determination, we have since developed a more automated method of determining erythrocyte speeds from EMV (automated tracking videos available)^31^.

We focused this manuscript on erythrocyte speed as opposed to overall retinal blood flow due to potential differences in vessel caliber in measuring vessel diameter using fluorescein and using ICG angiography, which were both used to determine vessel caliber in this study. Vessel diameter is an important component of blood flow determination, and future studies will be designed to take this into account. EMV sessions were generally conducted at the same time of day and it is possible that measurements could have been affected by diurnal variation. For individuals with glaucoma, it is possible that worsening of the disease in between imaging sessions could affect erythrocyte velocity measurements. Other limitations include the relatively small sample size and the varied diagnoses of this cohort that may affect retinal blood speed. While we did compare the intravisit variability in controls versus glaucoma and found no statistically significant difference, this sample was not large enough to study those differences in a robust manner. Future studies will explore changes in retinal blood flow with different conditions and disease states, such as glaucoma.

With the continued development of noninvasive methods of determining ocular blood flow, EMV may represent a reliable method for determination of retinal blood flow. The potentially accurate and reproducible measurement of erythrocyte speed further allows for determining the effect of disease states or pharmacologic agents on ocular blood flow – even if small in magnitude.

## Data Availability

The dataset generated and analyzed during the current study is available from the corresponding author on reasonable request.
